# The Role of Astrocytes in Neuroprotection after Brain Stroke: Potential in Cell Therapy

**DOI:** 10.3389/fnmol.2017.00088

**Published:** 2017-04-03

**Authors:** Andrea Becerra-Calixto, Gloria P. Cardona-Gómez

**Affiliations:** Cellular and Molecular Neurobiology Area, Group of Neuroscience of Antioquia, School of Medicine, Sede de Investigación Universitaria (SIU), University of AntioquiaMedellín, Colombia

**Keywords:** astrocytes, cell therapy, neuroprotection, cerebral ischemia, excitotoxicity

## Abstract

Astrocytes are commonly involved in negative responses through their hyperreactivity and glial scar formation in excitotoxic and/or mechanical injuries. But, astrocytes are also specialized glial cells of the nervous system that perform multiple homeostatic functions for the survival and maintenance of the neurovascular unit. Astrocytes have neuroprotective, angiogenic, immunomodulatory, neurogenic, and antioxidant properties and modulate synaptic function. This makes them excellent candidates as a source of neuroprotection and neurorestoration in tissues affected by ischemia/reperfusion, when some of their deregulated genes can be controlled. Therefore, this review analyzes pro-survival responses of astrocytes that would allow their use in cell therapy strategies.

## Introduction

**FIGURE 1:**
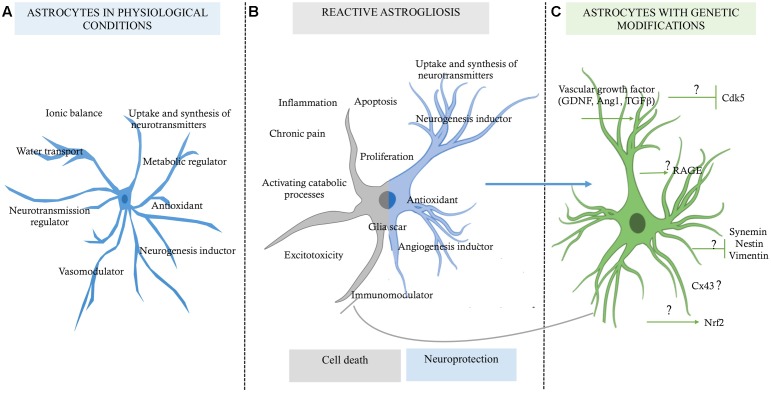
**Role of astrocytes in a micro-environment dependent-mode. (A)** Functions of the astrocytes in physiological conditions, which are in favor of the homeostasis of the nervous tissue. **(B)** Reactive astrocytosis, which has a double function highly discussed, one for cell death and one for pro-neuroprotection probably in a context dependent-mode. **(C)** Astrocytes with genetic modifications by reduced expression of some upregulated genes, which would allow preserve them as a neuroprotective source for promoting neuronal survival; although the mechanism of how they could maintain this state of neuroprotection for longer time is still unknown (?).

Until a few decades ago, astrocytes were considered to be glial support cells, with roles limited to providing trophic, metabolic, and structural support to the neurons. Currently, multiple investigations have revealed the multi-faceted role of astrocytes in cerebral parenchymal homeostasis, which depends on intercellular communication (Dallérac et al., 2013). It has been widely reported that astrocytic cells play protective roles in the nervous system, characterized by the ion buffering (Walz, 2000; D’Ambrosio et al., 2002), the uptake and synthesis of neurotransmitters (Danbolt, 2001; Kaczor et al., 2015), controlling cerebral blood flow (Abbott et al., 2006; Newman, 2015), transport of water (Zeng et al., 2007; Kitchen et al., 2015), release of antioxidant substances (Pentreath and Slamon, 2000), and immunomodulation (Dong and Benveniste, 2001). Also, it has recently been described that astrocytes are involved in adult neurogenesis (Mori et al., 2005; Buffo et al., 2008; Duan et al., 2015; Nato et al., 2015), making the astrocyte a highly complex cell (**Figure 1A**). However, under pathological conditions, hyperreactive astrocytes exacerbate its heterogeneous functions, presenting an opposite role that, contribute to the central nervous system (CNS) disbalance (**Figure 1B**).

Astrocytes are characterized as having a stellate morphology, which changes to a reactive state under stress and degenerative conditions. This individual morphology is directly related to the expression of glial fibrillary acidic protein (GFAP; Lebkuechner et al., 2015); the upregulation of GFAP depends on the nature of damage, the distance between the astrocyte and the site of injury, and the time after injury (Eng et al., 2000; Sun et al., 2010). On the other hand, it has been reported that reactive astrogliosis and astrocyte proliferation also are neuroprotective, because on this condition the glial cells can to provide factors that promote cell survival against severe injury or degeneration (Mohn and Koob, 2015; **Figure 1B**). However, under ischemia context, for example, the interruption of blood supply to the brain results in deprivation of oxygen and glucose and the consequent reduction of the energy supply. This process leads to excessive accumulation of ions and the deregulation of several signaling pathways, overloading the buffering role of the astrocytes, which go to favor of the activation of catabolic processes mediated by proteases, lipases, and nucleases, which disrupt neuronal function and lead to cell death (Lipton, 1999). Therefore, a better understanding of the astrocyte’s mechanism for triggering cell cytotoxicity or keeping its neuroprotective activity would help to control the parenchyma dyshomeostasis and to prevent progressive brain degeneration. In this review, we will focus on the cellular mechanism of the astrocytes for providing neuroprotection, and we will propose that the downregulation of proteins associated with pathological states could allow maintenance of its survival activity in the injured tissue, recovering its integrity and function.

## Astrocytes in Neurodegeneration After Brain Stroke

Astrocytes, unlike neurons, are less vulnerable to glutamate excitotoxicity during a brain stroke, however, induces its proliferation and increase GFAP levels in a process termed reactive astrogliosis (Sofroniew and Vinters, 2010). Susarla et al. (2014) reports that reactive protoplasmic astrocytes in the cortex began to proliferate within 3–5 days after injury, and the half of them reentering to the cell cycle up to a week later. Reactive astrogliosis have been related widely as a pathological hallmark of altered CNS tissue (Igarashi et al., 1999; Chen and Swanson, 2003; Rodríguez et al., 2009; Sofroniew and Vinters, 2010; Robel et al., 2015; Sofroniew, 2015). Which are generally found in areas surrounding severe focal lesions, and it is characterized by astrocyte proliferation and a considerable extension of processes beyond the previous domains of individual astrocytes (Swanson et al., 2004). These changes can result in reorganization of tissue architecture without the formation of dense narrow and compact barriers, as in glial scars (Sofroniew and Vinters, 2010; Shimada et al., 2011). The glial scar is the most characteristic profile of the reactive astrogliosis. In a severe state, a compact glial scar formation, includes changes associated with milder forms, with upregulation of GFAP and other genes, and pronounced hypertrophy of cell bodies and processes, interacting with other type of glial cells (Silver and Miller, 2004; Sofroniew and Vinters, 2010). In addition, glial scar formation has been considered an inhibitor of axon regeneration, as a maladaptive phenomenon that unavoidably causes neurotoxicity, inflammation, or chronic pain, supported by the increase of pro inflammatory factors levels, as interleukins 1β, 6, 10 (IL-1, IL-1β, IL-6, IL-10), interferon-γ (IFN-γ), transforming growth factor-β (TGFβ) (Rostworowski et al., 1997; Suzumura et al., 2006), reactive oxygen species (ROS), nitric oxide (NO), glutamate, calcium-binding protein B (S100b) (Piani et al., 1993; Pekny and Nilsson, 2005; Mori et al., 2009; Liu et al., 2011; Shen et al., 2012; de Pablo et al., 2013; Jiang et al., 2013). Also, producing molecules associated to infection and metabolic disorders, which exacerbate the disease, as Toll-like receptors (TLRs), lipopolysaccharides (LPS), β-amyloid (βA; Hu and Van Eldik, 1999; Caso et al., 2007; Gorina et al., 2011; Sofroniew, 2014), and ammonium (NH4; Lichter-Konecki, 2008). In brain stroke, reactive astrocytosis is related with “chronic cystic infarcts,” characteristic finding in a cortical cystic infarct is the preservation of the relatively paucicellular molecular layer overlying it and located immediately beneath the meninges, which then develops a dense collection of astrocytes, usually with gemistocytic phenotype. Astrocytic processes usually traverse the cystic cavity left by the death of brain tissue, in a delicate meshwork, with persistence of foamy histiocytes among and influx of polymorphonuclear leukocytes and macrophages (Sofroniew and Vinters, 2010). In the counterpart, there is a specific process of reactive astrogliosis that has beneficial functions and does not do harm. As describe below, many studies using transgenic and experimental animal models provide evidences that reactive astrocytes protect CNS tissue by the same mechanisms that astrocytes has homeostatic functions, as well as the induction of neurogenesis, regulation of immune system and maintain the blood–brain barrier (BBB) integrity. This evidence changes astrocytosis, from being viewed as harmful to being neuroprotective, while pathogenic expressions are being regulated (**Figure 1**).

## Support of Energy Metabolism and Antioxidant Effects of Astrocytes

Brain is a high energy-consuming organ; for these reason, brain cells can efficiently to utilize various energy substrates in addition to glucose, including lactate, pyruvate, glutamate, and glutamine (Bé Langer et al., 2011). Neurons and astrocytes present different metabolic profiles, although are complementary. However, astrocytes are reported that has an exclusive characteristic of support the neuron energetic needs. One of recently reported is the lactate metabolism represent an import pathway of glucose metabolism (Lovatt et al., 2007). Astrocytes present a high glycolytic rate, so enzymes as 6-phosphofructose-2-kinase/fructose-2,6-bisphosphatase-3 (Pfkfb3) are related to the activation of glycolytic pathway, involving the lactate as result of glucose oxidation. Those enzymes have high expression levels in astrocytes, but it is absent in neurons due to constant proteasome degradation (Herrero-Mendez et al., 2009). Another characteristic of astrocytes is the low expression levels of aspartate/glutamate carrier (AGC), which is a component of maleate aspartate shuttle, which operates the transfer of reducing equivalents from the cytosol to mitochondria (Berkich et al., 2007). In this context the conversion of derived pyruvate to lactate in cytosol ensure the maintenance of high NAD^+^/NAD ratio, being essential to sustain a high glycolytic rate (Bé Langer et al., 2011).

Another characteristic of astrocytes is the glycogen metabolism. Glycogen is the largest energy reserve of the brain and it can be rapidly metabolized under anaerobic conditions. Glycogen has been found to be almost exclusively localized in astrocytes in the adult brain (Brown, 2004), this characteristic suggest the close interaction between astrocytes and neurons. Brown et al. (2005) demonstrated that increasing astrocytic glycogen stores preserves the neuronal function and viability under limited conditions of energy, such as in hypoglycemia. Also, neuron–glia metabolic coupling involves glycogen content under the dynamic control of neurotransmitters and the neural functions (Brown et al., 2005). Gibbs et al. (2006) demonstrated that pharmacological inhibition of glycogenolysis in astrocytes using 1,4-dideoxy-1,4-imino-D-arabinitol (DAB), a potent inhibitor of glycogen phosphorylase, interrupts memory consolidation in young chickens in a bead discrimination learning task. Also, the role of astrocytic glycogen-derived lactate in long term memory formation, and for the *in vivo* maintenance of long-term potentiation (LTP) of synaptic strength in the mammalian brain (Suzuki et al., 2011). These findings support that the astrocytes play a major role in the metabolic maintenance of neurons, which are directly related to its functionality.

Additional characteristic of astrocytes is the role in the synthesis, re-uptake or recycling of neurotransmitters. It is widely known that astrocytes rapidly remove neurotransmitters that that are released in the synaptic cleft. This function is an essential process to guarantee the effective synaptic process and the maintenance of neuronal excitability. One of most important excitatory neurotransmitter is glutamate, its overstimulation is highly toxic for neurons, the way that astrocyte uptake the glutamate is through specific glutamate transporters named glutamate transported 1 (GLT-1), and glutamate aspartate transporter (GLAST) (Bak et al., 2006) in a glutamate–glutamine cycle (Shen et al., 1999; Danbolt, 2001). Astrocytes are responsible for replenishment of brain glutamate and are the only cell type in the brain which express pyruvate carboxylase, an enzyme involved in the anaplerotic pathway in the brain, effectively allowing them to synthesize glutamate from glucose (Bélanger and Magistretti, 2009; Bé Langer et al., 2011).

In brain stroke, the decrease of oxygen and glucose, produce an alteration of the glutamate levels, producing excitotoxicity in neurons. The evidence shows that GLAST and GLT-1 are downregulated shortly following the insult, which then precipitates glutamate-mediated excitotoxic conditions (Yi and Hazell, 2006; Zou et al., 2010). Also, Zou et al. (2010) demonstrated that glutamine synthetize (GS) inhibition in astrocytes significantly impaired glutamate uptake, suggesting that GS in astrocytes may represent a novel target for neuroprotection against neuronal dysfunction. Liang et al. (2008) compared the expression of excitatory amino acid transporters (EAATs) in astrocytes and microglia, analyses demonstrated that astrocytes express a much larger amount of membrane localized EAATs than microglia. Astrocytes prevented excito-neurotoxicity by the reduction of exogenous glutamate, whereas microglia did not. Conversely, activated microglia released an excess of glutamate that induced excitotoxic neuronal death. Astrocytes rescued neurons from microglial glutamate-induced death in a ratio-dependent manner. Inhibition of EAATs abolished glutamate uptake and the neuroprotective effect of astrocytes, but it did not alter microglial neurotoxic or neuroprotective effects. These results revealed that astrocytic EAATs can counteract microglial glutamate-induced neuronal death, whereas microglial EAATs are not involved in the toxicity and protection of astrocytes and microglia in a co-culture system (Liang et al., 2008). Those findings suggest that maintaining the expression and regulation of the transporters in astrocytes would be a potential source of clinical intervention treatment following brain ischemia.

On the other hand, astrocytes are involved in the defense against oxidative stress. Stroke produces several factors that contribute to increased brain vulnerability to oxidative stress, including its high rate of oxidative energy metabolism (an inevitable process generating ROS), and its high unsaturated fatty acids content (which are prone to lipid peroxidation; Dringen, 2000). Glutathione (GSH) is the most abundant antioxidant molecule in the brain, which acts directly as ROS scavenger or can be used as substrate for glutathione peroxidase (Dringen, 2000). GSH is regenerated for action of glutathione reductase, using NADPH as an electron donor; this process is essential for the maintenance of GSH in its reduced form (Bé Langer et al., 2011). NADPH is more abundant in astrocytes than in neurons, and astrocytes have a higher basal activity rate and a better capacity to stimulate this pathway in response to oxidative stress (Garcia-Nogales et al., 2003). Also, astrocytes release the antioxidant molecule ascorbic acid in response to glutamatergic activity. This ascorbic acid is taken up by neurons and modifies the local energy metabolism by inhibition of glucose consumption and increased uptake of lactate (Castro et al., 2009). Moreover, there is another molecule that is expressed in astrocytes and it has neuroprotective functions, the redox-sensitive transcription factor, named nuclear factor erythroid 2-related factor 2 (Nrf2) activation. Nrf2, is a redox-sensitive transcription factor, that coordinates expression of genes required for free radical scavenging, detoxification of xenobiotics, and maintenance of redox potential (Shih et al., 2005). In stroke, Nrf2 pathway is activated in both *in vitro* and *in vivo* ischemic models. In addition, to mediate self-defense in neurons, Nrf2 also actively regulates the expression of cytoprotective enzymes in other cell types within the neurovascular unit (NVU), including astrocytes and endothelial cells (ECs), and thus supports neuronal function and survival through cell–cell interaction (Yang et al., 2016).

A large body of experimental evidence suggests that astrocytes have a greater metabolic plasticity than neurons. A striking example is the differential response of astrocytes and neurons following the inhibition of mitochondrial respiration induced by NO. Astrocytes respond to NO with an increase in glucose metabolism through the glycolytic pathway, limiting the fall in ATP levels and preventing apoptosis. In neurons, however, this response does not seem to be present, and a similar NO challenge causes a massive ATP depletion, leading to apoptosis (Almeida et al., 2001; Bé Langer et al., 2011).

## Astrocytes Modulate the Immune Response

In ischemic conditions, reactive astrocytes are involved in the immune response because astrocytes mediate and propagate inflammatory signals in the brain, influencing various physiological and behavioral responses. Astrocytes were the first CNS cell type where was demonstrated the expression of class II major histocompatibility complex (MHC) molecules (Dong and Benveniste, 2001). MHC II is a molecule that play a critical role in the induction of immune responses through the presentation of processed antigens to CD41 T-helper cells, this molecule is normally expressed on professional antigen presenting cells (APCs), such as B cells, macrophages, dendritic cells, and other cell types, including astrocytes. MHC II expression allow to astrocytes can be regulated by cytokines, neurotransmitters, and neuropeptides (Dong and Benveniste, 2001). Although, the MHC is involve in an exacerbated inflammation response in astrocytes. Also, cytokines have neuroprotective and neurotrophic roles required for neurodevelopment and maintenance of normal CNS function (Maier et al., 2005; Morganti-Kossmann et al., 2007).

As mentioned before astrocytes are also capable of synthesizing cytokines and chemokines; they express pattern-recognition receptors (PRRs), such as TLRs, scavenger receptors, and complement proteins. There is evidence that astrocytes play a complex and a dual role in the local regulation of immune reactivity. Astrocytes are resistant to apoptosis induced after inflammation by death receptors, which is known as apoptosis antigen 1 and tumor necrosis factor (TNF)-related apoptosis-inducing ligand (FAS, TRAIL), indicating that these cells are well prepared to survive under inflammatory insults (Farina et al., 2007). Neurotrophic mediators such as glial-monocyte colony stimulating factor (GM-CSF), vascular endothelial growth factor (VEGF), neurotrophin 4 (NT-4), and ciliary neurotrophic factor (CNTF) can activate TLRs in astrocytes and stimulate restorative pathways (Bsibsi et al., 2006).

In an ischemic condition, activated microglia and activated astrocytes (reactive astrogliosis), might to secret products exerting neuroprotective actions during glial scar formation. It has been demonstrated that reactive astrocytes and microglia can to demarcate the damaged area and to limit the leukocyte extravasation, promoting BBB repair and neuronal survival (Farina et al., 2007). Some cytokines, such as IL-1 and IL-6, related to pathological inflammatory responses in different processes might also behave as a mediator of neuroprotection. Some studies have demonstrated that deletion of IL-6 and IL-1β increases BBB permeability and decreases the production of neurotrophic factors such as CNTF and insulin growth factor (IGF), indicating that cytokine-induced astrogliosis following trauma is important to restore the integrity of the BBB and to repair the lesion (Herx et al., 2000; Mason et al., 2001). Several *in vitro* data demonstrate that cytokines such as IL-1, IL-6, and TNF support the production of neuroprotective mediators. Other proteins implicated in inflammation response, are the chemokines, which facilitate the mobilization of leukocytes and polymorphonuclear cells. Additionally, these chemokines contribute to the migration of neural progenitors in the developing brain (Tran and Miller, 2003) and toward areas of brain injury (Belmadani et al., 2006; Farina et al., 2007). These characteristics make the astrocyte a cell highly sensitive, inductor and possibly regulator of immune responses, being one of main important characteristic in an ischemic context, because the chronicity of inflammation depends on the degree of tissue damage and exacerbation of the injury.

## Neuroprotective Role of Astrocytes

Under a loss of cerebral parenchymal integrity, astrocytes would play a protective role whether is possible to control its reactivity (Li et al., 2008), which would allow maintaining the homeostatic functions. In particular, astrocytes are involved in a large number of key processes in the nervous system, including crucial roles in regulating vascular tone; removing excess glutamate in synaptic cleft thus limiting the neuronal activity; promoting synaptogenesis; releasing neurotrophic factors; secreting different antioxidants and responding to the release of pro and anti-inflammatory molecules (Sofroniew and Vinters, 2010). Astrocytes have also been shown to regulate the blood flow during neuronal activity, via the release of vasoactive substances such as NO, products derived from activity of epoxygenase, ATP and cyclooxygenase, activation of phosphatidylinositol 3-kinase (PI3K) and calcium waves propagated from the neuron to astrocyte and endothelium (blood vessel cells; Abbott et al., 2006), and regulate potassium ions [K^+^] resulting from synaptic activity (Benarroch, 2005; Bean, 2007). Concomitant with the ionic and metabolic regulation by astrocytes, these cells may regulate the release of neurotrophic factors, which facilitate neuronal survival and angiogenesis (Goss et al., 1998; Rz et al., 1999; Jiang et al., 2013). These neurotrophic factors have varied effects on neurons, ECs, microglia, leukocytes, and neural stem cells (NSCs) (Schwartz and Nishiyama, 1994; Igarashi et al., 1999; Swanson et al., 2004; Oliveira et al., 2013; Götz et al., 2015).

## Astrocytic Involvement in Adult Neurogenesis

The adult neurogenesis in the adult mammalian brain has been described in two specific zones, named neural niches; the subgranular layer (SGL) of the dentate gyrus (DG) of the hippocampus and the subventricular zone (SVZ) in the lateral wall of the lateral ventricle (Seri et al., 2001). The primary precursors in the SVZ, the other germinal region of the adult brain, have been identified as having the characteristics of astrocytes and expressing GFAP (Laywell et al., 2000). The reports are suggested that some of these cells can maintain a neurogenic potential and act as NSCs (Mori et al., 2005; Magnusson and Frisén, 2016). These cells can divide and generate new neurons under normal conditions or after the chemical removal. Seri et al. (2001) found GFAP^+^ cells called type D cells are derived from the astrocytes and probably function as a transient precursor in the formation of new neurons. However, deeper research is needed to understand the trans-differentiation of astrocytes in NSC or a transient state as trophic source without a definitive commitment to other specific linage.

After brain ischemic event, astrocytes acquire stem cell hallmarks mainly *in vitro* conditions; *in vivo* is still controversial because the multipotentiality is depending of growth factors (Götz et al., 2015). *In vitro* assays have shown that some of the reactive astrocytes in the traumatic or ischemic brain acquire neurosphere (NS)-forming ability, multipotency and long-term self-renewal, while others remain within their astrocyte lineage *in vivo* (Götz et al., 2015). Jiang et al. (2013) found two different types of cells that are NPC-astroglial cells (NPC-Astros) and Olig2PC-astroglial cells (Olig2PC-Astros), respectively. When was grafted into brains subjected to global ischemia, Olig2PC-Astros exhibit neuroprotective effects and improved behavior (Jiang et al., 2013). On other hand, Ohab et al. (2006) reported that stroke induces neurogenesis from a GFAP-expressing progenitor cell in the SVZ and migration of newly born neurons into a unique neurovascular niche in peri-infarct cortex. Also, Faiz et al. (2015) described that reactive astrocytes can convert to multipotent NSCs capable of NS formation and multi-lineage differentiation *in vitro*. Moreover, they reported that SVZ NSCs give rise to a reactive astrocytes subpopulation in the cortex that contribute to astrogliosis and scar formation and they found that these astrocytes in SVZ can be converted to neurons *in vivo* by forced expression of Ascl1 (Faiz et al., 2015).

Astrocytes also preserve the function of the hippocampal neural niches, where adult neurogenesis plays an important role. Sultan et al. (2015) found that astrocytes can allow the synaptic integration of adult-born hippocampal neurons, allowing local dendritic spine maturation, which is necessary for the *N*-methyl-D-aspartate receptor (NMDAR)-dependent functional integration of newborn neurons. Moreover, Tao et al. (2015) demonstrated that some proteins as β-arrestin-1 (β-arr1) is secreted by astrocytes in DG and participates in adult hippocampal neurogenesis. The model of β-arr1 knockout (KO) astrocytes produced less NSs, and RNA-sequencing revealed that β-arr1 KO DG astrocytes exhibit an aberrant gene expression profile of niche factors, including elevated transcription of Bmp2. Those results suggest that β-arr1-mediated nuclear signaling regulates the production of excretive factors derived from niche of astrocytes and expansion of neural precursors in DG, thus maintaining homeostasis of adult hippocampal neurogenesis (Tao et al., 2015). Additionally, Li et al. (2014), in an oxygen glucose deprivation/reperfusion (OGD/R) model, examined the levels of a damage-associated molecular-patterning molecule called high-mobility group box 1 (HMGB1) that is release by astrocytes. HMGB1 is critical for NSC/neuroprogenitors proliferation during brain development. The data demonstrate that HMGB1 released by OGD/R astrocytes promotes NSC proliferation through activation of the PI3K/protein kinase B (PI3K/Akt) signaling pathway (Li et al., 2014). These findings demonstrate that astrocytes are involved key roles in the generation of new neurons and maintenance, stimulation of neural niches after an ischemic event, and possibly in the regulation of adult neurogenesis in physiological conditions, which do them targets for isolation, study and cell therapeutic cells in this type of brain damage. However, some characteristic of NSC associate with astrocytes still is controversial, hence their neurogenic potential must be further explored.

## Angiogenesis can be Modulated by Astrocytes

The appropriate supply of oxygen and glucose, as well as the appropriate secretion of metabolites by the brain, depends on close relationships between the vascular system, glial cells, and neurons. The interconnection between these cells is known as the NVU. Astrocytes have very close interactions with neurons and ECs, which composite the blood vessels. In this interaction participate various membrane proteins such as ion and water channels and receptors for growth factors and cytokines. Also, astrocytes and ECs can release neurotrophins, vascular growth factors, glucose, and amino acids, in order to generate stability and maintenance of the BBB (Abbott et al., 2006).

During brain ischemia, there is a series of events that compromise the integrity of the BBB. Glucose deficit and hypoxia trigger cellular stress, not only in neurons, but also in ECs and astrocytes. This cellular stress causes the release of oxygen, free radicals, and NO; and also the production of pro-inflammatory cytokines that allow permeabilization of the BBB and therefore infiltration of leukocytes, which exacerbate the inflammatory response and increase the triggering of apoptotic signals (Chodobski et al., 2011). In this condition, signaling between astrocytes, pericytes, and endothelium become disrupted. Hence, repairing the gliovascular system include a crosstalk between astrocytes and pericytes (Bell et al., 2010). Astrocytes are known to release thrombospondin-1, which is a major regulator of synaptic maturation (Christopherson et al., 2005). Reactive astrocytes also release tissue plasminogen activator, which may be required for recovering neurons to remodel their dendritic arbors (Xin et al., 2010). Indeed, several studies have suggested that the therapeutic benefits of stem cell therapies may depend in part on the ability of astrocytes to amplify the effects on neuronal remodeling (Shen et al., 2010). Astrocytes and cerebral ECs secrete many trophic factors that support oligodendrocyte precursor cells (Arai and Lo, 2009). And after injury, VEGF-mediated endothelial recovery is linked with the proliferation and migration of oligodendrocyte precursor cells (Le Bras et al., 2006; Hayakawa et al., 2011).

Moreover, astrocytes has different mechanism to modulate cytotoxic response, as inducing angiogenesis necessary for the reestablishment of the blood circulation after brain ischemia. This mechanism consist of the secretion of a range of chemical agents and glia-derived factors, including TGFβ, glial-derived neurotrophic factor (GDNF), basic fibroblast growth factor (bFGF), angiopoietin 1 (Ang1) (Igarashi et al., 1999; Wuestefeld et al., 2012). These factors can stimulate the production of new blood vessels and the proliferation of endothelial progenitor cells (EPCs; Cross et al., 2001; Babaei et al., 2003). Different studies using co-cultures of brain ECs and astrocytes have demonstrated that astrocytes express functional receptors for a high proportion of agents that mobilize ECs (Abbott et al., 2006). Additionally, reactive astrocytes can release factors, as HMGB1, VEGF, Ang1, and angiotensin 2 (Ang2) (Hayakawa et al., 2012; Duan et al., 2015; Shen et al., 2016); which promotes EPC-mediated neurovascular remodeling during stroke recovery (Krum and Rosenstein, 1998). Hayakawa et al. (2011, 2012), found that blocking the “receptor for advanced glycation end products” (RAGE) increased the reactive astrocyte and ECs significantly decreased EPC-endothelial adherence. RAGE is a multi-ligand receptor that propagates cellular dysfunction in several inflammatory disorders, tumors and diabetes, and it is upregulated at sites where its ligands are accumulated; being low expressed in normal tissues. Also, it has been reported that RAGE may play a dual role in the inflammatory response, because interaction of RAGE on leukocytes or ECs with its ligands results in cellular activation involving the transcription factor nuclear factor kappa-light-chain-enhancer of activated B cells (NF-kappaB). On the other hand, RAGE on ECs may function as an adhesive receptor that directly interacts with leukocyte ss2-integrins (Chavakis et al., 2004). So, through the upregulation of RAGE on affected ECs, the reactive astrocytes may augment EPC adherence and transmigration. This phenomenon may comprise a novel mechanism whereby crosstalk between reactive astrocytes and cerebral endothelium augments EPC responses for neurovascular recovery in damaged or diseased brain. In this order, RAGE is important in EPC induction and adherence that can play an important role in tissue vascularization and endothelium homeostasis after CNS damage by ischemia. Those findings suggest that astrocytes can partially regulate the effects of ischemia through a continue communication with NVU cells.

## Possible Proteins Involved in Astrocytes Reactivity and Regeneration

As described above, astrocytes appear to possess a high potential for regeneration and neuroprotection following stroke, but also there are substantial evidence that has shown that astrocytes are cells that exacerbate ischemic injury (**Figure 1B**).

So, one possible paradigm of astrocyte reactivity control is the expression of GFAP. Increased GFAP expression is associated with hyperreactivity and altered morphology of the astrocyte. Also, connexin-43 (Cx43) is a gap junction protein that is associated with reactive astrogliosis. In response to an acute needle stab wound *in vivo*, reactive astrocytes expressed Cx43 in the peripheral zone surrounding the injury site (Theodoric et al., 2012). In addition, intermediate filament proteins such as vimentin, nestin, and synemin are involved in the reactivity of astrocytes and are highly expressed in conditions of deprivation of oxygen and glucose, as well as in response to oxidative stress (de Pablo et al., 2013). However, the regulatory mechanisms underlying the development of reactive astrocytes during basal function and under neuroprotective hypertrophic function remain unknown.

Other protein involved in the reactive astrocyte that has been poorly studied is cyclin-dependent kinase 5 (CDK5). This protein is highly expressed in post-mitotic neurons. In the brain, it is involved in functions such as neuritogenesis, synapse formation, synaptic transmission, and the assembly, organization and stabilization of the axonal cytoskeleton, and it allows the activation of apoptosis. Its substrates are mainly neurofilaments, microtubule-associated proteins (MAPs) and elements of the axonal cytoskeleton (Grant et al., 2001). Although CDK5 is generally studied in neurons, it is not exclusively expressed in those cells; some reports show that CDK5 is active in non-neuronal cells such as astrocytes, ECs, and blood cells (Liebl et al., 2011). In astrocytes, CDK5 has a very important activity in the process of elongation and reactivity. He et al. (2007) report that p35, GFAP, and CDK5 can form immunocomplexes in primary cultures of astrocytes. p35 is activated when the astrocyte is subjected to stress caused by injury, generating active CDK5. Astrocytes treated with roscovitine, a CDK5 inhibitor, show reorganization of tubulin and GFAP (He et al., 2007). In addition, other findings have shown that CDK5 plays a significant role in the regulation of EC proliferation (Liu et al., 2008). Therefore, CDK5 dysregulation in excitotoxic processes can generate changes not only in neurons but elsewhere in the NVU, which is highly regulated by astrocytes. This in turn leads to increased reactivity and alteration of each of the components and the integrity of the NVU and results in brain dysfunction.

## Could Genetically Modified Astrocytes be a Cell Therapeutic Strategy for Cerebral Ischemia?

Due to the complexity of the excitotoxic effects caused by ischemia/reperfusion, in which pharmacological treatments have only palliative effects or require administration within a short time period after injury (van der Worp and van Gijn, 2007), it is necessary to find alternative therapies focused on long-term neuroprotection. Cell therapy in neuroscience is focused on the search for cells that can induce regeneration, providing a vehicle for corrective molecular systems to trigger endogenous cellular events that can restore tissue homeostasis in progressive neurodegeneration. Currently, many therapeutic strategies using astrocytes have been focused primarily on diseases associated with the spinal cord, such as amyotrophic lateral sclerosis (ALS; Nicaise et al., 2015). In addition, astrocyte transplantation leads to recovery of axonal myelination, modulation of the immune response and release of neurotrophic factors that prevent oxidative stress and excitotoxic damage (Choudhury and Ding, 2015).

Transplantation models have been reported in cerebral ischemia mainly using mesenchymal stem cells (MSC; Hsuan et al., 2016; Lee et al., 2016), induced pluripotent stem cells (iPSCs; Mohamad et al., 2013), and NSCs (Huang et al., 2014; Gervois et al., 2016), showing that there is stimulus of endogenous activity of cells in the injured tissue, generating increased angiogenesis/neovascularization and release of trophic factors, which is chiefly secreted by these cells, allowing the migration to the lesion zone. Likewise, there is axonal recovery, dendritic branching, and synaptogenesis, which are reported mainly surrounding the injury site (Horie et al., 2015). Interestingly, the intravenous NSC administration improves the gliotransmission property, which induced the excitatory–inhibitory balance in the penumbra area (Bacigaluppi et al., 2016).

**FIGURE 2:**
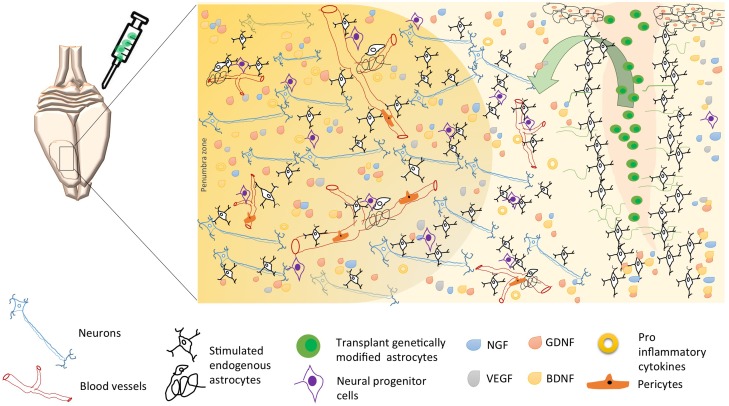
**Possible mechanisms of neuroprotection induced by astrocytes’ cell therapy after brain stroke.** Following brain ischemia, transplantation of genetically modified astrocytes could trigger endogenous neuroprotective mechanisms as neurogenesis, angiogenesis, and regulation of inflammation. These mechanisms are regulated by a paracrine activity, where the astrocyte participates as an intermediary between neurons, endothelial cells, pericytes, microglia, and neural progenitor cells; through the release of neurotrophic factors (BDNF, GDNF, VEGF, GDNF), lower proinflammatory cytokines, and increased reuptake excitatory neurotransmitters, thus avoiding excitotoxicity and, promoting long-term neuronal survival. Arrow, soluble growth factors release by transplanted genetically modified astrocytes (“green cells,” e.g., **Figure 1C**), stimulate endogens astrocytes and endothelial cells, preserving the neurovascular integrity.

Other reports have proposed astrocytes as a therapeutic target based on their control by genetic modification of proteins related to the immune response and to the exacerbation of reactivity and cytotoxicity (Choudhury and Ding, 2015; Merienne et al., 2015). We and other researchers have found that Tau hyperphosphorylation is increased by focal and global cerebral ischemia, which is closely related to spatial memory impairment (Gutiérrez-Vargas et al., 2015), implying the alteration of proteins that regulate microtubule assembly, such as glycogen synthase kinase 3 (GSK3; Céspedes-Rubio et al., 2010) and CDK5 (Rashidian et al., 2009; Gutiérrez-Vargas et al., 2016), and remodeling of the actin cytoskeleton, such as the small GTPases RhoA and Rac (Rho family of GTPase members; Posada-Duque et al., 2015b). These proteins have therefore been suggested as key targets in the pathogenesis and recovery of the cerebrovascular unit after infarction (Posada-Duque et al., 2015a, targeting not only neurons but also astrocytes (Posada-Duque et al., 2015b). In the case of CDK5, we have designed a shRNAmiR that, in a cerebral ischemia model, resulted in neurologic and motor improvement during the first week after ischemia. Additionally, CDK5 RNAi (RNA interference) prevented dysfunctions in learning, memory and reversal learning at a month after ischemia. Interestingly, silencing CDK5 decreases the hyperreactivity of astrocytes at 1 month post-treatment (Gutiérrez-Vargas et al., 2015) but increased astrocytic arborization under neuroprotective conditions at 4 months post-ischemia (Gutiérrez-Vargas et al., 2016). In addition, our *in vitro* assays have shown that CDK5 RNAi in astrocytes generates stellation and brain-derived neurotrophic factor (BDNF) release in a mechanism dependent on active Rac, providing neuroprotection in co-cultures of astrocytes and neurons (Posada-Duque et al., 2015b). However, it has been widely reported that astrocytes have limited neuroprotective capacity, primarily because of their hypertrophic reactivity under neurotoxic and neurodegenerative processes. This activity involves cytoskeletal changes mediated by Rho GTPases such as Rac/RhoA and by CDK5, as well as the participation of these cells in glial scar formation, the increase of free radicals, and the pro-inflammatory response that prevents neuronal plasticity (Gleichman and Carmichael, 2014). Our most recent findings validated the potential of CDK5 knock-down astrocytes transplanted in ischemic rats, which surprisingly generated motor function recovery, branching of endogenous astrocytes and increased EC adhesion by PECAM1 and Ki67^+^ cells in SVZ after 1 month post transplantation (Becerra-Calixto and Cardona-Gómez, 2016). This functional effect was maintained for 4 months and astrocytes recovery the EC adhesion and transendothelial electrical resistance (TEER) affected by glutamate in a co-culture *in vitro* model (unpublished data). Therefore, these results lead to the proposal of cell therapy based on silenced astrocytes for genes associated with hyperreactivity (**Figure 1C**). Based on our evidence, CDK5 knock-down astrocytes may provide neuroprotective functions by inducing the activation of endogenous surrounding astrocytes, which would trigger paracrine signaling or activity of another cell population of the NVU. Transplantation of these modified astrocytes also mobilizes neural progenitor cells, which, combined with their other protective effects, could increase survival and neuroprotection mainly in the penumbra areas of infarcted tissue, recovering neurological function (**Figure 2**).

## Conclusion and Perspectives

The field of neuroscience is currently focusing on the study of the function of non-neuronal cells, which seems to play a key role, especially in the survival of neurons upon cerebral injuries. However, it remains highly discussed how and when astrocytes may provide neuroprotection. Several authors indicate that there are different types of astrocytic populations with effects on neuroprotection, neurogenesis and regulation of the immune response. Thus, the next challenge for validating the use of astrocytes in cell therapy is to explore the specific profile of neuroprotective astrocytes.

Furthermore, cell therapy has some limitations, such as maintaining long-term restoration, potential regenerative abilities of some types of cells, the capacity of mobilization toward the injured tissue and their safe use in humans. These limitations necessitate further analysis and discussion before the clinical application of those cells. Therefore, further studies must be performed to elucidate how to use astrocytes as targets for cell therapy in acute and chronic neurodegenerative diseases.

## Author Contributions

AB-C wrote the manuscript, GC-G did critical revision of the manuscript for intellectual content. All authors participated in the study concept and design, read and approved the final manuscript.

## Conflict of Interest Statement

The authors declare that the research was conducted in the absence of any commercial or financial relationships that could be construed as a potential conflict of interest. The reviewer PFC and handling Editor declared their shared affiliation, and the handling Editor states that the process nevertheless met the standards of a fair and objective review.

## Abbreviations

AGC: aspartate/glutamate carrier
ALS: amyotrophic lateral sclerosis
Ang: angiopoietin
APCs: antigen presenting cells
βA: β-amyloid
BBB: blood–brain barrier
BDNF: brain-derived neurotrophic factor
bFGF: basic fibroblast growth factor
BMEC: brain micro-endothelial cells
CDK5: cyclin-dependent kinase 5
CNTF: ciliary neurotrophic factor
Cx43: connexin-43
DAB: 1,4-dideoxy-1,4-imino-D-arabinitol
DCX: doublecortin
DG: dentate gyrus
EAATs: excitatory amino acid transporters
ECs: endothelial cells
EPCs: endothelial progenitor cells
GDNF: glial-derived neurotrophic factor
GFAP: glial fibrillary acidic protein
GLAST: glutamate aspartate transporter
GLT-1: glutamate transported 1
GM-CSF: glial-monocyte colony stimulating factor
GS: glutamine synthetize
GSH: glutathione
GSK3: glycogen synthase kinase 3
HMGB1: high-mobility group box 1
IGF: insulin growth factor
IL: interleukin
iPSCs: induced pluripotent stem cells
KO: knockout
LPS: lipopolysaccharides
LTP: long-term potentiation
MAPs: microtubule-associated proteins
MSC: mesenchymal stem cells
NH4: ammonium
NMDAR: *N*-methyl-D-aspartate receptor
NO: nitric oxide
NPs: neural progenitors
Nrf2: nuclear factor erythroid 2-related factor 2
NS: neurosphere
NSCs: neural stem cells
NT-4: neurotrophin 4
NVU: neurovascular unit
OGD/R: oxygen glucose deprivation/reperfusion
PECAM1: platelet and endothelial cell adhesion molecule 1
Pfkfb3: 6-phosphofructose-2-kinase/fructose-2,6-biphosphatase-3
PRRs: pattern-recognition receptors
RAGE: receptor for advanced glycation end products
RhoA and Rac1: Rho family of GTPase members
ROS: reactive oxygen species
S100b: calcium-binding protein B
SGL: subgranular layer
siRNA: small interference Ribonucleic acid
SVZ: subventricular zone
TGFβ: transforming growth factor-β
TLRs: Toll-like receptors
TRAIL: TNF-related apoptosis-inducing ligand
VEGF: vascular endothelial growth factor.
